# Privacy-preserving data sharing infrastructures for medical research: systematization and comparison

**DOI:** 10.1186/s12911-021-01602-x

**Published:** 2021-08-12

**Authors:** Felix Nikolaus Wirth, Thierry Meurers, Marco Johns, Fabian Prasser

**Affiliations:** Berlin Institute of Health at Charité – Universitätsmedizin Berlin, Charitéplatz 1, 10117 Berlin, Germany

**Keywords:** Biomedical data sharing, Privacy, Usefulness, Systematization, Distributed computing, Secure multi-party computing, Data enclave

## Abstract

**Background:**

Data sharing is considered a crucial part of modern medical research. Unfortunately, despite its advantages, it often faces obstacles, especially data privacy challenges. As a result, various approaches and infrastructures have been developed that aim to ensure that patients and research participants remain anonymous when data is shared. However, privacy protection typically comes at a cost, e.g. restrictions regarding the types of analyses that can be performed on shared data. What is lacking is a systematization making the trade-offs taken by different approaches transparent. The aim of the work described in this paper was to develop a systematization for the degree of privacy protection provided and the trade-offs taken by different data sharing methods. Based on this contribution, we categorized popular data sharing approaches and identified research gaps by analyzing combinations of promising properties and features that are not yet supported by existing approaches.

**Methods:**

The systematization consists of different axes. Three axes relate to privacy protection aspects and were adopted from the popular Five Safes Framework: (1) safe data, addressing privacy at the input level, (2) safe settings, addressing privacy during shared processing, and (3) safe outputs, addressing privacy protection of analysis results. Three additional axes address the usefulness of approaches: (4) support for de-duplication, to enable the reconciliation of data belonging to the same individuals, (5) flexibility, to be able to adapt to different data analysis requirements, and (6) scalability, to maintain performance with increasing complexity of shared data or common analysis processes.

**Results:**

Using the systematization, we identified three different categories of approaches: distributed data analyses, which exchange anonymous aggregated data, secure multi-party computation protocols, which exchange encrypted data, and data enclaves, which store pooled individual-level data in secure environments for access for analysis purposes. We identified important research gaps, including a lack of approaches enabling the de-duplication of horizontally distributed data or providing a high degree of flexibility.

**Conclusions:**

There are fundamental differences between different data sharing approaches and several gaps in their functionality that may be interesting to investigate in future work. Our systematization can make the properties of privacy-preserving data sharing infrastructures more transparent and support decision makers and regulatory authorities with a better understanding of the trade-offs taken.

## Background

### Introduction

Data sharing is the practice of making data from research and healthcare available for secondary purposes and to third parties. This enables data-driven medical research, which promises to significantly improve public health as well as prevention, diagnosis, treatment and follow-up care [1, 2]. It is advocated at both national and international levels [3–5] and is steadily becoming a standard practice in biomedical research [6]. The benefits of data sharing include larger sample sizes and the ability to generate new insights and to replicate results in times of increasing personalization of medicine. Data sharing is also associated with higher citation rates [7, 8] and promoted by several funding agencies [9, 10].

Despite its promises, several obstacles make data sharing difficult and often even impossible. An important obstacle are legal issues [11], caused by severe restrictions on the processing of personal medical data imposed by national and international data protection laws. Important examples include the US Health Insurance Portability and Accountability Act (HIPAA) [12] and the EU General Data Protection Regulation (GDPR) [13]. To process data in compliance with these regulations, organizational and legal procedures need to be implemented to protect the privacy of patients and research participants. An important prerequisite for data processing in medical research is usually informed consent. However, collecting consent can be difficult and is not always feasible [14], in particular when data is to be shared retrospectively at large scale. An alternative that is often suggested (and permitted in many jurisdictions) is anonymization, i.e. the altering of data in such a way that individual patients and research participants cannot be identified, rendering the data non-personal [15]. However, a trade-off between privacy protection and the quality and hence utility of output data needs to be considered in this process [16]. In this context, the complexity and heterogeneity of clinical and research data makes effective anonymization without disproportionately negative effects on data quality sometimes difficult and in some cases even impossible [17]. How strict the requirements for anonymization are depends on the applicable legislation. For example, while the HIPPA Privacy Rule [12] provides an interpretable and implementable framework, anonymization under the GDPR is more difficult due to a lack of concrete requirements and resulting heterogeneous policies and legal interpretations [18]. In addition, researchers often do not want to lose control of their data and institutions are often reluctant to disclose data that is considered confidential from a business perspective, e.g. for competitiveness reasons [19].

These challenges can be tackled by implementing infrastructures that enable analyzing data stored in distributed databases and computing a common result without exchanging individual-level data [10]. In the context of this work, we refer to such methods as “data sharing infrastructures”, which involve different parties or sites (e.g. hospitals) in a joint analysis.

On the methodological side, there are different options for implementing this process. One well-known example is the exchange of aggregated statistics (see e.g. [20]), which are then combined to a common result, comparable to a meta-analysis. Another example is cryptographic protocols (e.g. [21]), enabling different parties to jointly process a function on their private data without revealing each other’s input. Such modern secure multiparty computing schemes often employ homomorphic encryption, which supports operations such as addition and multiplication on encrypted data [22].

Technology infrastructures built on these approaches have already been successfully used to investigate a range of medical questions. Examples include studies of associations of maternal movement and newborn birth size [23], outcomes of partial or full knee replacement [24], treatment patterns for comorbidities of patients suffering from cancer [25], survival of patients with intrahepatic cholangiocarcinoma [26] and of interactions between food intake as well as gut bacteria and metabolite patterns [27]. Other projects have implemented manual processes for distributed data analysis, such as the 4CE consortium [28], which focuses on the clinical trajectory of COVID-19 patients or a study carried out in the German Medical Informatics Initiative, focusing on multi-morbidity and rare diseases [29].

### Objectives and contributions

Despite the fact that privacy-preserving infrastructures are often considered to be the most important enabler for comprehensive data sharing in the medical domain and despite the multitude of technological approaches available and studies that have successfully utilized such technologies (see above), these infrastructures are only rarely used for sharing healthcare and medical research data today. We believe that one of the main reasons for this is uncertainties for decision makers and regulatory authorities regarding the exact characteristics of such infrastructures, particularly regarding the degree of privacy protection and anonymity for data subjects they provide. Indeed, as we will show in this article, there are fundamental differences between current solutions.

As a first step towards making the properties of data sharing infrastructures more transparent, the aim of this work is to introduce a systematization of general techniques and their properties along two dimensions. Firstly, the systematization is intended to structure the design space, as a development step towards tools for comprehensively assessing the privacy protection properties of data sharing infrastructures. Secondly, we also believe that the systematization can contribute to developing instruments for assessing the usefulness of data sharing infrastructures, i.e. the impact that their protection mechanisms have on options to analyze data compared to the simple (but often not feasible) approach of pooling all data in a common database.

The need for a framework for comparing different approaches to data sharing is also illustrated by the fact that several previous papers have been published on related topics (see section “Comparison with prior work”). However, our work is fundamentally different in that we do not only consider specific types of solutions (e.g., based on cryptographic methods) and aim at systematically mapping the usefulness dimension in addition to the privacy protection dimension. This comes at the expense of a higher degree of abstraction.

To show that our approach is practicable, we used it to perform a high-level analysis and comparison of several existing solutions. In summary, our work provides the following contributions:

1. We present a high-level and technology-agnostic framework consisting of three axes describing the degree of protection and three axes describing the degree of usefulness provided by data sharing infrastructures.
2. We use this framework to analyze and compare ten different real-world data sharing platforms. Our results show that they can be grouped into three general types of solutions with common properties.
3. Based on our results we derive insights into research gaps that may be worthwhile to investigate when developing next-generation data sharing infrastructures.

## Methods

### Trade-off between privacy protection and usefulness

Data sharing would be easy to implement if all relevant data could simply flow freely and be stored in a common database. As mentioned above, this is not possible in practice, however. Any attempt to take measures to meet privacy protection requirements inevitably leads to limitations in comparison to this basic approach. These limitations may relate, for example, to the time that the data sharing process takes or to the number of analysis methods supported. This fundamental conflict between unrestricted processing of data and the protection of the privacy of data subjects is well known in the field of privacy-enhancing technologies. An important example is data anonymization, where, as also mentioned above, the quality of output data often must be traded off against the degree of privacy protection achieved (see e.g. [30]).

**Fig. 1:**
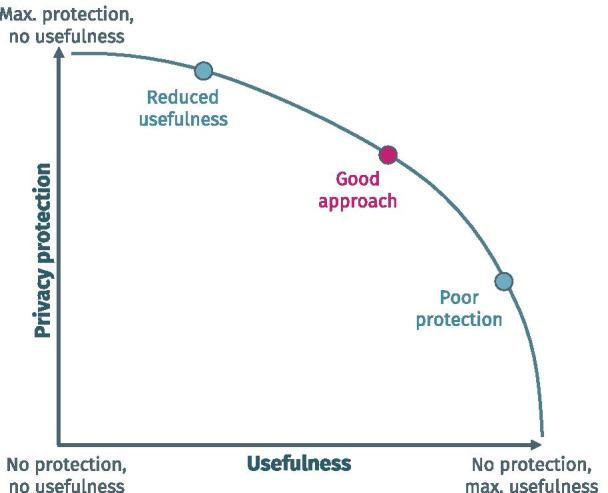
Abstract graph illustrating the trade-off between the degree of privacy protection and the usefulness of a data sharing infrastructure

Similar trade-offs must be made when designing and implementing privacy-preserving data sharing infrastructures. Figure 1 provides an abstract, schematic illustration of this trade-off. It is derived from the concept of risk-utility curves, as used in data anonymization research (see e.g. [31]). The y-axis describes the level of privacy protection, while the x-axis describes the level of usefulness of an infrastructure. Examples of aspects that could be captured by the x-axis include the spectrum of functionalities offered, how scalable their implementations are and how much work is required to add new functionalities.

There are two extreme types of approaches. Approaches located in the top-left corner significantly limit the amount of data shared, e.g. only patient or research participant counts, which typically implies a very high degree of protection. Approaches located in the bottom-left corner exchange fine-grained data in nearly unmodified form, e.g. by pooling all data in a central database which is open for access by researchers. Obviously, this would be extremely useful, but offers little privacy protection.

In between these two extremes, there is a broad spectrum of potential solutions based on different trade-offs between privacy protection and usefulness. To be relevant, those data sharing approaches need to provide added value in comparison to the basic approaches, i.e. they need to significantly reduce privacy risks, while maintaining a high degree of usefulness. In the graph, this is indicated by the non-linear relationship between the extreme points.

One example is the aforementioned meta-analysis approach in which more than counts can be exchanged when appropriate safeguards are implemented (e.g. for regression coefficients [32]). Still, functionality is limited, as only aggregated data from individual sites can be included in the analysis, hence reducing the number of (scientific) questions that can be answered. At the same time, privacy is relatively easy to protect by making sure that the aggregate data released does not leak sensitive personal information.

### A framework for systematizing properties of data sharing techniques

**Fig. 2:**
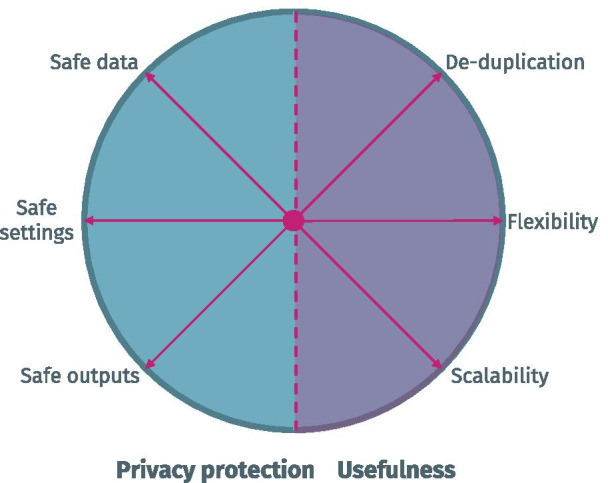
Illustration of privacy protection and usefulness axes considered

For assessing the degree of privacy protection and the usefulness provided by data sharing approaches, we propose a first systematization containing three axes for each of these aspects. These axes are illustrated in Fig. 2 and will be explained in more detail in this section.

### Aspect 1: Assessing the degree of privacy protection provided

As a baseline for assessing the degree of protection provided we suggest to apply the Five Safes Framework, which was developed by Desai, Ritchie and Welpton as a general framework for reasoning about privacy protection when sharing data [33] (important examples are discussed in Section “Comparison with prior work”).

**Fig. 3:**
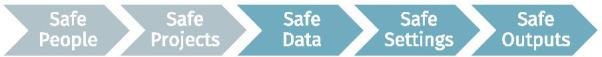
Elements of the five safes framework. Axes with relevance to this work are highlighted in blue

The Five Safes Framework specifies five different axes, which are illustrated in Fig. 3: (1) Only *Safe People*, e.g. trustworthy researchers, should be provided with access to data (cf. the British Office for National Statistics research and data access policy [34]), (2) only *Safe Projects* should be carried out, e.g., analyses that respect patient privacy and which are appropriate from an ethical perspective, (3) only *Safe Data* should be processed meaning that identifiability should be reduced to an acceptable minimum already on the level of input data (cf. the principle of data minimization under the GDPR and the Minimum Necessary Standard of HIPAA [10]), (4) *Safe Settings* should be used for providing access or performing analyses, which reduces the likelihood that sensitive data is leaked during processing and (5) *Safe Outputs* should be guaranteed (e.g., by ensuring that the output of analyses does not disclose sensitive personal information).

For our framework we will only consider the technical aspects of the Five Safes Framework and thus exclude the first two axes, *Safe People* and *Safe Projects*. The reason is that these aspects need to be either addressed on an organizational level (e.g. ethics committee / Institutional Review Board (IRB) approval) or with technical solutions that are not directly related to data sharing (e.g. Authentication and Authorization Infrastructures). In the context of data sharing, there are specific measures that can be taken along the remaining technical axes:

#### Axis 1.1: Safe data

Data provided as input to analyses supported by data sharing is considered safe if the identifiability of patients or research participants has been reduced. *Safe Data* can for example be obtained by anonymization, aggregation or encryption. Protection achieved with the first two techniques may be irreversible, while it may be possible to decrypt encrypted data at the end of the process. Even if anonymization or aggregation has limitations, residual risks of identifiability can potentially be managed by implementing safeguards along the other axes.

#### Axis 1.2: Safe settings

The setting in which distributed data is processed is considered safe if no or at least only some data is leaked during processing. A well-known example of a *Safe Setting* are virtual data access environments, in which data can be analyzed without handing out individual-level data, e.g. through a remote desktop connection. Infrastructures using cryptographic secure multi-party computing protocols also provide a secure setting in which data can be analyzed in an encrypted form only and only mutually calculated results can be decrypted [35] (more details will be provided in the “Results” section). However, even with such safe settings being used to perform analyses, additional efforts may need to be made to ensure that the results are also safe.

#### Axis 1.3: Safe outputs

The result calculated using a data sharing infrastructure is considered safe, if the resulting data disclosed to the users of the infrastructure is non-identifiable/non-personal. One way of achieving this is to only allow computations producing aggregate data. However, this must be carefully designed, as e.g. disclosing statistical tables with small cell counts can reveal details about individuals [36]. To mitigate this risk, anonymization methods can be used to transform data before it is being disclosed. For example, data points can be rounded up, they can be omitted or random noise can be added [37]. A state-of-the-art technique to provide *Safe Outputs* is Differential Privacy which formulates a general mathematical property for data processing algorithms that, if parameterized correctly, renders output data non-identifiable [38]. We note that a data sharing infrastructure will automatically provide *Safe Outputs* when *Safe Data* is provided as input (cf. the meta-analysis approach).

### Aspect 2: Assessing the usefulness of data sharing technologies

As a first step, we suggest to assess the usefulness of infrastructures for sharing medical data in terms of three different axes that reflect important requirements in multi-institutional medical research: (1) *De-duplication*/*record-linkage*, which refers to the ability to combine data from different sources while taking into account that some records might relate to one another (e.g. to the same patient), (2) *Flexibility*, which reflects the degree to which a solution is able to support different types of statistical analyses and use cases as well as adapt to different analytical requirements as they can change over time and (3) *Scalability*, that refers to how an infrastructure performs when the amount of data or the complexity of an analysis increases.

#### Axis 2.1: De-duplication/record-linkage

**Fig. 4:**
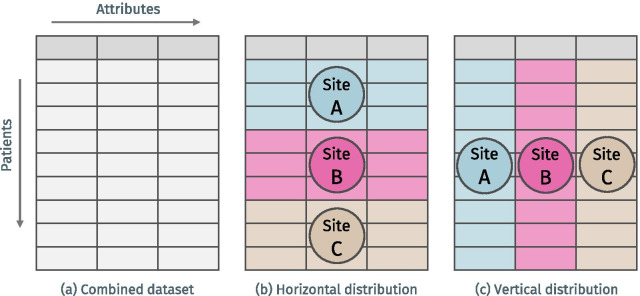
Horizontal and vertical data distribution

This axis is related to the ability to resolve different types of data distribution, which are sketched in Fig. 4. Most data sharing infrastructures are able to resolve horizontal distribution of data but ignore potential relationships on the level of individuals. This is for example the case with meta-analyses in which patient data from different hospitals is simply added to a larger sample without checking for population overlap. In order to determine or resolve such overlap, privacy-preserving methods for reconciling records belonging to the same individuals must be implemented, which is non-trivial. This becomes even more challenging, when also vertical distribution is to be resolved. A typical example is the need to integrate different types of data for the same patients stored at different locations (e.g. at hospitals and health insurances). Procedures allowing for such a cross-site duplicate resolution range from probabilistic linkage algorithms [39] and cross-site pseudonymization methods to secure linkage based on encrypted identifying information using secure multi-party computing protocols [40, 41]. This results in different characteristics with regard to risks and usefulness, which manifests itself, for example, in the possibility of verifying the correctness of linkage results. A cross-site pseudonymization procedure poses the greatest risks but provides the highest linkage quality, whereas probabilistic linkage and cryptographic methods offer a very high level of protection, but make it difficult to verify the results. The associated risks are reflected by axes 1.1, 1.2 and 1.3, while the usefulness of de-duplication and record-linkage is reflected by this axis. For the sake of clarity, we will simply refer to this axis as “*De-duplication*” in the remainder of this article.

#### Axis 2.2: Flexibility

This axis refers to the ability of infrastructures to support a range of different analyses and to its extensibility to future use cases. For example, some of the solutions analyzed in this article have been tailored towards a limited set of very specific functionalities (e.g. cohort selection). On the other hand, some solutions are based on generic frameworks that provide a high degree of extensibility and options to integrate new analysis methods. In between these two extremes, there are solutions, e.g. based on meta-analyses, which offer a certain degree of flexibility, but only support some types of analyses. For example, the quality of survival analyses might be inconsistent, analyses of subgroups might require additional efforts for each subgroup and longitudinal studies as well as explorative investigations and assessments of data quality may be difficult to perform [42, 43]. There are also differences regarding the effort required to integrate new methods into different types of solutions. For example, integrating new types of analyses into solutions based on secure multi-party computing requires developing implementations using special cryptographic primitives, which is time-consuming and requires expert-level knowledge of cryptography.

#### Axis 2.3: Scalability

This axis refers to the ability of an infrastructure to function well, i.e. to return a result to the analysis performed within a reasonable timeframe and with a reasonable demand for compute and storage resources, when load is increased (this is also called load scalability [44]). Within the context of data sharing infrastructures, an increase in load can be caused by an increase in the volume (e.g. number of patients) or dimensionality (e.g. number of attributes per patient) of the data analyzed, or the number of sites participating in the sharing process. *Scalability* is a particular challenge for approaches based on secure multiparty computing, as current state-of-the-art approaches are known to not scale well with respect to both of these aspects. It general, it can be said that the performance of all secure multiparty computing methods is determined by the number of messages exchanged between the parties involved, the required number of rounds of communication between the parties and the computational overhead per round. It should be noted, however, that the exact increase in computational complexity depends on the particular type of method used [45]and the operation performed [46].

## Results

In this section, we present the results of an application of the framework proposed for an analysis of a range of well-known data sharing infrastructures for medical data that exhibit different characteristics along the axes suggested. We note that some infrastructures are relatively generic and can be used to implement different methods with different characteristics. In these cases, we analyzed typical applications of the infrastructures and present alternative use cases in the “Discussion” section. In particular, we analyzed the following solutions: SHRINE/i2b2 [47], dataSHIELD [20], OMOP/OHDSI [48]*,* Personal Health Train [49], Clinerion Patient Network Explorer [50], TriNetX [51], MedCo [52], Sharemind MPC [53] and examples implementing the popular Data Enclave concept [54, 55]. Based on common privacy protection properties of the approaches studied, we assigned them to three different categories: (1) distributed data analysis, (2) cryptographic secure multi-party computing approaches and (3) data enclaves.

### Distributed data analysis

One category, termed distributed data analysis, contains approaches that exchange aggregated and potentially anonymized data only. This non-personal data is generated locally at the participating sites and then merged across locations using meta-analysis methods. Hence, regarding our framework, only aggregated or anonymized data (and thus *Safe Data*) is exchanged (axis 1.1), no *Safe Setting* is hence needed (axis 1.2) and *Safe Outputs* are provided by design (axis 1.3). However, there are significant limitations regarding the analytical utility of these types of data sharing approaches. None of the solutions analyzed from this category supports *De-duplication* (axis 2.1), since vertical integration can only be conducted with additional measures (see “Discussion” section). Moreover, some of the approaches in this category are very specific and others are quite generic (*Flexibility*, axis 2.2), while all share the disadvantages of meta-analyses described in section “Aspect 2: Assessing the usefulness of data sharing technologies”, such as limited possibilities to perform subgroup analyses. However, all approaches provide a high degree of computational *Scalability*, as computations can be offloaded to the participating sites effectively (axis 2.3). Important examples of approaches in this category are:

- *SHRINE/i2b2* Informatics for Integrating Biology & the Bedside (i2b2) is an open-source clinical data warehouse used in various projects worldwide [47]. The Shared Health Research Information Network (SHRINE) is an extension of i2b2 for distributed analysis [56]. It allows the creation of a network of peer sites, in which aggregated results of queries are collected. SHRINE is for example used in a registry of pediatric patients with rheumatic disease [57] or in a network supporting clinical trial recruitment [58]. The solution is specific, since it has been designed specifically to support cohort selection functions (*Flexibility*, axis 2.2).
- *DataSHIELD* This software supports distributed analyses based on the R statistical computing environment [20]. It creates a network of server nodes that connect to local instances of R. Through a client node, researchers can then send commands which are distributed to the local sites to calculate aggregated results without individual-level data leaving the sites. DataSHIELD has been deployed, for instance, in a network that investigates interactions of ageing, mental well-being and environment [59] and it is a generic solution, since it supports a range of analysis methods based on R (*Flexibility*, axis 2.2).
- *OMOP/OHDSI* The Observational Health Data Sciences and Informatics (OHDSI) project [48] has developed the Observational Medical Outcomes Partnership (OMOP) Common Data Model (CDM), which can be used to create highly structured and standardized local databases for real-world evidence studies. For distributed analyses, scripts can be executed at the sites to derive aggregate data that can then be combined in meta-analyses. This process is also supported by a range of tools provided by the OHDSI community. In practice, this approach has for example been utilized in a study of models for predicting stroke in women [60]. The European Health Data Evidence Network (EHDEN) [61] aims to foster the adoption of OMOP/OHDSI in Europe. The approach is generic, since a wide range of analyses is supported (*Flexibility*, axis 2.2).
- *Personal Health Train* The Personal Health Train (PHT) is data sharing concept developed by different private and public contributors [49]. It is based on a train analogy: (1) the data sources are called train stations, (2) the data analysis methods (e.g. query and merge procedures) are called trains. In all current implementations only aggregated data leave the stations towards the trains, hence implementing a meta-analysis approach. The PHT has for instance been used to realize a study on distributed learning for predicting the post-treatment two-year survival of lung cancer patients [62]. It is a generic solution conceptualizing a container-based data sharing infrastructure that can be used to implement wide a range of meta-analysis approaches (*Flexibility*, axis 2.2).
- *Clinerion Patient Network Explorer* and *TriNetX* Both the Patient Network Explorer by Clinerion [50] and the software by TriNetX [51] are parts of propriety data sharing networks for hospitals established by these companies. After installing the software, local nodes in the hospitals provide interfaces for central services to collect aggregated data, for instance the number of patients meeting certain inclusion criteria. As an example, TriNetX has been used to collect data for investigating the risk of COVID-19 for people suffering from intellectual and developmental disabilities [63]. Both solutions can be described as specific, as privacy protection is implemented by restricting the analysis methods supported (*Flexibility*, axis 2.2).

### Secure multi-party computation

Approaches using cryptography-based secure multi-party computation protocols to ensure that only encrypted individual-level data leaves the participating sites form an important additional category of data sharing infrastructures. Typically, it is also ensured that only analytical results aggregating the data from multiple sites can be decrypted at the end of a computation (thus also providing protection on the institutional level). As a result, only *Safe Data* (i.e. encrypted data) is exchanged (axis 1.1) in a *Safe Setting*, as data is not disclosed during processing (axis 1.2). All solutions identified that fall into this category further implement specific analysis methods that ensure that only *Safe Outputs* are disclosed (axis 1.3). We note, however, that this is not an inherent property of cryptographic approaches but a result of performing secure analyses or perturbing output data by the approaches investigated. It is well-known that *Scalability* can be a problem for secure multi-party computation protocols (axis 2.3). Performance is often non-linear in the number of participating sites, implementations require a lot of computational resources and low-latency network connections with a high transmission rate, which can typically not be provided when data is shared over the internet. Whether or not duplicates can be detected and resolved (*De-duplication*, axis 2.1) and different types of analyses can be performed (*Flexibility*, axis 2.2) depends on the exact implementation. Important examples of approaches from this category are:

- *MedCo* The open source software MedCo uses additively homomorphic encryption to enable researchers to perform analyses on encrypted data across sites [52]. The analysis results are encrypted and can only be decrypted by authorized investigators. MedCo is implemented as an extension to i2b2 (analogously to SHRINE). The software, for example, forms the backbone of the SCOR network for sharing data on patients with COVID-19 [64]. The software focuses on cohort exploration and survival analysis. MedCo does not support resolving duplicates (*De-duplication*, axis 2.1) and is specific, as it only supports a limited set of functionalities and extensions require implementations to be developed based on the cryptographic methods used by the software (*Flexibility*, axis 2.2).
- *Sharemind MPC* This proprietary software has been developed by the company Sharemind. Similar to MedCo it enables computations on encrypted data hosted at multiple sites without decrypting it first [53]. The software is oriented towards data scientists. Analyses can either be designed in a proprietary programming language or in an environment which resembles the R statistics programming environment. The solution has, for example, been used to analyze 10 million synthetic health records distributed to 1,000 health centers that also involved detecting and removing duplicates (*De-duplication*, axis 2.1) [65]. Sharemind MPC provides a generic framework for privacy-preserving data sharing (*Flexibility*, axis 2.2).

### Data enclaves

The third category of approaches consists of implementations of the data enclave concept, in which individual-level data of one or multiple sites is submitted to a data custodian maintaining a secure environment for data access [66]. Eligible researchers can run queries against the data stored by the custodian, the results of which are checked for anonymity before they are returned. Hence, individual-level, non-safe data is exchanged (*Safe Data*, axis 1.1) but access is restricted through a *Safe Setting* (axis 1.2) which ensures that no data is leaked and that output data is safe (*Safe Outputs*, axis 1.3). On the usefulness dimension, duplicate resolution is supported (*De-duplication*, axis 2.1) and large datasets as well as data from many participant sites can be shared in scalable manner (*Scalability*, axis 2.3). However, real-world implementations differ regarding their extensibility and *Flexibility* (axis 2.2). Important examples of data enclaves are:

- *Scottish National Safe Haven* This enclave is operated by the Scottish National Health Services (NHS) and provides access to various health datasets [54]. Data is stored in pseudonymized form to enable record linkage. Data access is provided through a virtual network with no internet access and no ability to install custom software. The infrastructure has, for example, been used to study temporal trends in breast cancer incidence [67]. The solution is somewhat generic, as typical data analysis methods are supported, but extensibility is limited as additional software, packages and functionalities can only be implemented by the enclave (*Flexibility*, axis 2.2).
- *US Center for Medicare and Medicaid Services Virtual Research Data Center* This enclave is operated by the US Center for Medicare and Medicaid Services and provides access to claims data combined with other types of medical data [55]. To ensure that output data is safe, researchers are only allowed to export aggregated information which is reviewed and screened for identifiability before it can be downloaded [68]. The system has, for example, been used for a study on the relative risk of Alzheimer’s disease among patients with prostate cancer who received androgen deprivation therapy [69]. The solution is specific, since its software and functionalities focus on integration and analysis of claims data (*Flexibility*, axis 2.2).

## Discussion

### Principal results

In the previous sections, we have proposed a schema for systematizing privacy-preserving data sharing infrastructures for medical research. We applied this framework to study a wide range of solutions proposed and found that they can be assigned to three distinct categories, based on common properties. Table 1 summarizes the results of our analysis.

**Table 1:** Results of our analysis of solutions for privacy-preserving data sharing

Approach	Year of publication	Category	1. Privacy protection			2. Usefulness		
			1. Safe data	2. Safe settings	3. Safe outputs	1. De-duplication	2. Flexibility	3. Scalability
SHRINE/i2b2	2008	Distributed data analysis	Yes	No	Yes^b^	No	Specific	Yes
dataSHIELD	2010	Distributed data analysis	Yes	No	Yes^b^	No	Generic	Yes
OHDSI	2014	Distributed data analysis	Yes	No	Yes^b^	No	Generic	Yes
Personal Health Train	2017	Distributed data analysis	Yes	No	Yes^b^	No	Generic	Yes
Clinerion	2015	Distributed data analysis	Yes	No	Yes^b^	No	Specific	Yes
TriNetX	2015	Distributed data analysis	Yes	No	Yes^b^	No	Specific	Yes
MedCo	2018	Secure multi-party computation	Yes^a^	Yes	Yes	No	Specific	No
ShareMIND	2008	Secure multi-party computation	Yes^a^	Yes	Yes	Yes	Generic	No
Scottish National Safe Haven	2015	Data enclave	No	Yes	Yes	Yes	Generic	Yes
Virtual Research Data Center	2014	Data enclave	No	Yes	Yes	Yes	Specific	Yes

^a^The processed data is encrypted individual-level data and thus safe

^b^*Safe Outputs* is an implicit result of providing *Safe Data* as input

As can be seen from this summary, most solutions identified fall into the category of distributed data analyses. One reason for this could be the fact that the technical complexity of this approach is relatively low, while it supports a fairly wide range of use cases. In comparison, secure multi-party computation is quite complex from a technical perspective and data enclaves are difficult to set up in some legislations, as individual-level data may not be allowed to leave the institutions in which it was initially collected. Distributed data analysis, however, reaches its limits when analyses on individual-level are needed or complex record-linkage and duplicate detection functionalities are required. Secure multi-party computation and data enclaves are relatively new approaches to medical data sharing, which can provide more functionalities. For them to be used even more widely, technical challenges (e.g. regarding suitable cryptographic protocols) as well as legal challenges (e.g. regarding the question whether encrypted data be considered non-personal or what an appropriate legal status for data custodians could look like) will need to be overcome. To accelerate work on these issues, policymakers should consider incentives for making innovative choices regarding data sharing architectures.

### Comparison with prior work

Our work builds on the Five Safes framework to systematize privacy protection. In prior work, the framework has already been used to study data sharing in official statistics [70], social and political sciences [71] and psychology [72]. In the biomedical domain, the framework has been adopted to model risk-based anonymization approaches [73]. To the best of our knowledge, our work is the first to apply the framework to common biomedical data sharing infrastructures, however. Moreover, we have complemented the Five Safes framework for modeling privacy protection with additional axes for systematizing the usefulness of data sharing technologies, considering common requirements from biomedical research. Other articles analyzing data sharing infrastructures, such as the work by Foster [71], are not systematic and do not focus on biomedical research.

Other frameworks for data sharing in biomedical research have been proposed, which can also be used to analyze different technical approaches. These focus on other aspects, however. For example, Knoppers [74] proposed a framework for the sharing of genomic data with a particular emphasis on trust, responsible research and oversight using organizational and legal safeguards. This is comparable to the non-technical axes *Safe People* and *Safe Projects* of the Five Safes Framework [33]. Moreover, Aziz et al. [75] presented an overview of privacy-preserving techniques for sharing genomic data, which is particularly sensitive and difficult to protect from privacy breaches. Hence, the paper puts a specific focus on cryptographic methods tailored towards genomic data sharing, which provide strong and provable degrees of protection. Compared to our approach their framework used for comparisons is rather specific, focusing on cryptographic algorithms and their technical properties and less on off-the-shelf, more generic infrastructures. Still, many of the aspects used by Aziz et al. in their comparisons are partially congruent to aspects of our framework (e.g. execution time, memory usage and network communication as aspects of *Scalability*, secure computations and output privacy as synonyms for *Safe Settings* and *Safe Outputs*, and accuracy as an aspect of *Usefulness*), which can be seen as an additional indicator for the broad applicability of our framework. Also Mittos et al. [76] presented a systematization of privacy-enhancing technologies for processing genomic data. However, their work focuses on many different types of processing, from which data sharing is just one example. Still, many of the open issues identified, such as the computational costs of some approaches and the need to improve the usefulness of results are in-line with our findings. Naveen et al. [77] presented an overview of applications, challenges and solutions for genomic data processing, which also includes aspects of data sharing. Their work contains lists of known privacy threats and specific approaches for implementing different use cases while mitigating those threats. Along these lines they systematically analyze open challenges within different application areas, but do not propose a common systematization spanning all of them. Notably, they also highlight some of the challenges mentioned in our work, such as the inherent trade-off between degrees of protection and usefulness. Thapa et al. [78] presented an overview of data sharing technologies for the more general area of “precision health”, also focusing primarily on cryptographic methods and methods requiring specific hardware support (e.g. Trusted Computing Environments). Consequently, the aspects used in their comparison of different approaches are quite similar to the aspects used by Aziz et al., which are well aligned with our more high-level framework as discussed above. In addition to that, they analyzed specific applications of data sharing frameworks, e.g. for distributed machine learning. The axes used for comparing such solutions could serve as a basis for future extensions of our framework (see section “Limitations, future work and open research questions”).

A framework for real-world multi-database studies has been presented by Toh [79]. On a conceptual level, this framework is most closely related to our work. However, it puts a strong focus on study design and feasibility and thus only considers weighing analytic flexibility with privacy protection on the utility and risk axes as well as trading off data pooling and distributed analyses on the technology axes. Finally, a comprehensive, yet unsystematic, overview of infrastructures for sharing data on COVID-19 has been presented by Raisaro et al. [64].

### Limitations, future work and open research questions

We note that the systematization proposed is abstract and of a qualitative nature. It is hence only suited for performing initial high-level comparisons of different solutions in the field as exemplified by the results of our analysis of selected implementations. Although a rigorous and formal framework would be desirable to enable more detailed comparisons, constructing such a framework is highly challenging. Important reasons can be found in a recent comment by Richie and Green [80] in which the authors advocate for the qualitative nature of the Five Safes framework. Aziz et al. [75] also report challenges in identifying technical and quantitative criteria that are general enough to apply to different types of approaches and that at the same time can be used for specific comparisons.

At a more fundamental level, even the quantitative modeling of privacy risks and usefulness is still an open research problem. Both aspects can only be captured by models that make very specific assumptions, which in turn may not apply to all projects and usage scenarios. For example, a recent overview by Wagner and Eckhoff lists 80 different formal privacy models [81]. However, some data sharing infrastructures and approaches support different privacy models to provide *Safe Data* and *Safe Outputs*, e.g. Differential Privacy [38] or solutions limiting the uniqueness of disclosed data, such as cell suppression [82] or k-anonymity [83]. In future work, we plan to extend our framework by incorporating the most common models. Regarding the usefulness of solutions, some of the more fine-grained axes used in [75, 78] might serve as a starting point. One example is *Accuracy*, which reflects the impact of privacy models on output data quality and hence captures the risk-utility trade-off inherent to such technologies.

The results of our analysis of the current landscape of solutions can also provide insights into potential directions for future work on data sharing methods. One important example is the low number of solutions supporting de-duplication or record linkage. When analyzing horizontally distributed data, the inability to identify and resolve population overlap can significantly reduce the quality of results [84]. If a study intends to analyze vertically distributed data, record linkage is crucial, as different data sets need to be combined on a patient-level. One important example is research on rare diseases, as patients with such conditions typically visit a wide range of healthcare providers and relevant data for each patient is therefore inherently distributed. Future work could be carried out to extend distributed data analysis infrastructures with record-linkage functionalities, e.g. by enriching data with secure record linkage tokens [85]. Also, secure multi-party computation environments could be extended with libraries including different record-linkage algorithms (see [86] for a recent example). Moreover, future work could explore ways to provide strong protection guarantees for inherently flexible approaches, such as the Personal Health Train. This could, for example, be achieved by integrating libraries providing support for a wide range of privacy-preserving analysis functions within such infrastructures. Finally, a challenge with privacy-preserving data sharing infrastructures is that access to individual-level data in some cases cannot be provided at all, although access to data from at least one site is often needed to develop analysis algorithms that can then be executed in the distributed network. One approach to overcome this limitation is to provide synthetic data derived from the original data for this preparatory process (see [87] for a recent example in the context of distributed data analysis).

## Conclusion

In this article, we proposed a high-level framework for analyzing and comparing privacy-preserving data sharing infrastructures for medical research. We believe that our framework makes the properties of data sharing approaches more transparent and can serve as a starting point for developing more comprehensive systematizations, ultimately supporting decision makers and regulatory authorities in gaining a better understanding of the trade-offs taken. We have shown that our systematization is of value, by using it to analyze existing solutions, showing that there are fundamental differences between them. Finally, our results also provide insights into gaps, regarding the systematization itself as well as the current landscape of data sharing infrastructures, that may be worth exploring in the future.

## Data Availability

All data generated or analyzed during this study are included in this published article.
